# Interaction of *growth* *hormone* *receptor/binding protein* gene disruption and caloric restriction for insulin sensitivity and attenuated aging

**DOI:** 10.12688/f1000research.5378.1

**Published:** 2014-10-28

**Authors:** Oge Arum, Jamal Saleh, Ravneet Boparai, Jeremy Turner, John Kopchick, Romesh Khardori, Andrzej Bartke

**Affiliations:** 1 Department of Internal Medicine, Southern Illinois University-School of Medicine, Springfield, IL, 62794, USA; 2 Department of Surgery, Division of ENT-Otolaryngology, Southern Illinois University-School of Medicine, Springfield, IL, 62794, USA; 3 Edison Biotechnology Institute and Department of Biomedical Sciences, Heritage College of Osteopathic Medicine, Ohio University, Athens, OH, 45701, USA; 4 Department of Internal Medicine, Division of Endocrinology & Metabolism, Eastern Virginia Medical School, Norfolk, VA, 23507, USA

**Keywords:** Longevity regulation; endocrinology & metabolism; insulin sensitivity; growth hormone hormonal signaling; caloric restriction; (neuro)endocrinology of senescence

## Abstract

The correlation of physiological sensitivity to insulin ( *vis-à-vis* glycemic regulation) and longevity is extensively established, creating a justifiable gerontological interest on whether insulin sensitivity is causative, or even predictive, of some or all phenotypes of slowed senescence (including longevity). The *growth* *hormone* *receptor/* *binding* *protein* gene-disrupted (GHR-KO) mouse is the most extensively investigated insulin-sensitive, attenuated aging model. It was reported that, in a manner divergent from similar mutants, GHR-KO mice fail to respond to caloric restriction (CR) by altering their insulin sensitivity. We hypothesized that maximized insulin responsiveness is what causes GHR-KO mice to exhibit a suppressed survivorship response to dietary (including caloric) restriction; and attempted to refute this hypothesis by assessing the effects of CR on GHR-KO mice for varied slow-aging-associated phenotypes. In contrast to previous reports, we found GHR-KO mice on CR to be *less* responsive than their *ad libitum* (A.L.) counterparts to the hypoglycemia-inducing effects of insulin. Further, CR had negligible effects on the metabolism or cognition of GHR-KO mice. Therefore, our data suggest that the effects of CR on the insulin sensitivity of GHR-KO mice do not concur with the effects of CR on the aging of GHR-KO mice.

## Introduction

Improvements in insulin sensitivity or blood glucose homeostatic management are hallmarks of many slow-aging mutant and dietarily restricted animals, supporting the conjectures that these endocrine and metabolic phenomena may be positive regulators of (or simply indicators of interventions that might promote) longevity [ Arum *et al.*, 2009; Bartke, 2008; Bonkowski *et al.*, 2006; Lawler *et al.*, 2008; Longo & Finch, 2003; Masoro, 2003; Masoro, 2005; Mattison *et al.*, 2007; Piper & Bartke, 2008]. A recently proffered approach to biomedical ventures endeavoring to delay aging, and thus increase healthspan (the period of life during which an organism is free of substantial morbidity or physiological disability/inability), begins with studying interventions that increase lifespan [ Kenyon, 2010; Miller, 2009; Olshansky *et al.*, 2007; Warner & Sierra, 2009]. Therefore, it is of high gerontological interest to study causal associations between longevity and physiological correlates that might result in anti-aging healthspan therapies based on engendering those physiological correlates, or that might serve as useful biomarkers for pharmacological or lifestyle interventions to delay the onset and/or decelerate the rate of senescence.

The *growth hormone receptor/binding protein (Ghr/bp)* gene-disrupted (knockout) (GHR-KO) mouse is homozygous for a targeted disruption (knock-out, KO) of the *growth hormone* (GH) *receptor* (GHR) */binding protein* gene, and is thus GH-resistant, resulting in decreased GH hormonal signaling. GHR-KO mice were generated by insertional mutagenesis that disrupted the *Ghr/bp* gene; this results in decreased hepatic production of insulin-like growth factor 1 (IGF-1), which leads to markedly reduced levels of circulating IGF-1, a reduced growth rate, an approximately 20% reduction in adulthood length, and an approximately 40% reduction in adult body weight [ Zhou *et al.*, 1997].

Of particular note, the GHR-KO mouse outlives its littermate control by approximately 40% [ Coschigano *et al.*, 2000; Coschigano *et al.*, 2003].

Produced in the β-cells of the pancreatic Islets of Langerhans, the hormone insulin regulates metabolism and energy homeostasis, partly by inducing the tissue uptake of glucose from blood. The GHR-KO mouse exhibits markedly decreased plasma insulin levels, due partly to decreased proliferation of β-cells [ Liu *et al.*, 2004]. As blood insulin concentration inversely mediates the systemic insulin sensitivity, insulin sensitivity is greater in the GHR-KO mouse than in its littermate control [ Liu *et al.*, 2004]. This is also the case with multiple other long-lived mice [ Brown-Borg *et al.*, 1996; Conover & Bale, 2007; Conover *et al.*, 2008; Dominici *et al.*, 2002; Selman *et al.*, 2008].

Results from survivorship studies reveal that aging-retarding (and thus, lifespan-increasing) dietary restriction (DR), including yet not limited to caloric restriction (CR), further increases insulin sensitivity and survivorship for some long-lived mutants, the Ames ( *Prop1 ^df/df^*) Dwarf mouse [ Bartke *et al.*, 2001] and the *growth hormone releasing hormone* KO ( *Ghrh ^-/-^*) mouse (data not shown). However it has been reported that CR doesn’t influence insulin sensitivity, and only modestly increases the survivorship of females, in the GHR-KO mouse [ Bonkowski *et al.*, 2006].

As an initial hypothesis, if CR fails to exert much effect on one senescence-associated trait (longevity) of the GHR-KO mouse because its level of insulin sensitivity is already as great as permissible for a viable animal, then a GHR-KO mouse on CR should not vary from a GHR-KO mouse on an *ad libitum* (A.L.) diet in other aging-associated characteristics (namely, metabolism and cognition).

Therefore, we attempted to investigate whether insulin sensitivity is sufficient to explain the severely attenuated response to CR of slow-aging associated phenotypes in GHR-KO mice. Surprisingly, in the course of our experiments we discovered that CR actually increases blood insulin concentration and starkly reduces insulin sensitivity in GHR-KO mice. These results question the assertion that CR has no effect on GHR-KO mouse blood glucose homeostatic management and the relationship, if any, between insulin sensitivity and slowed senescence in GHR-KO mice.

## Materials and methods

### Animal husbandry

***Ethics statement.*** Animal Protocol #178-02-001 was approved by the Laboratory Animal Care and Use Committee of Southern Illinois University-School of Medicine.

*Ghr/bp* gene-disrupted (GHR-KO) mice were generated by inserting a neomycin cassette replacing the 3′-end of the fourth exon and the 5′-end of intron 4/5 of the genomic sequence [ Zhou *et al.*, 1997]. The founder population of GHR-KO mice was provided by Dr. John J. Kopchick (Ohio University, Athens, OH). GHR-KO and GHR-N (heterozygous *littermate* controls for GHR-KO mice) mice were generated by mating of GHR-KO males with females heterozygous for the *Ghr/bp*-disrupted allele (GHR-N). These breeding schemes produce *littermate* control mice that have the same genetic background and are subject to the same intra-uterine and post-natal environment as the mutants.

Abiding by service provider’s instructions for sample collection and shipping, genotyping was conducted via quantitative polymerase chain reaction (q-PCR)-based technologies (Transnetyx, Inc., Cordova, TN).

The resulting mice had elements of a 129/Ola, a Balb/c, two C57Bl/6J, and two C3H/HeJ stocks; therefore, although lacking the methodological benefits of “reproducible genetic heterogeneity” [ Miller, *et al.*, 1999], this stock possesses considerable genetic variation, and thus the results are likely applicable to other mouse populations.

The animals were maintained in shoebox-type cages in light- (12 hours light to 12 hours darkness) and temperature- (22 ± 2ºC) controlled rooms with constant access to Lab Diet Formula 5001 (23% protein, 4.5% fat, 6% fiber) (Nestlē Purina, St. Louis, MO) and tap water. Littermate control pups were weaned at the age of 21–23 days, and GHR-KO pups two weeks later or at the time of weaning control pups from the next litter.

All experiments were performed in female mice, as GHR-KO stock male littermate controls are insensitive to the common dosage of insulin (0.75 U.S.P.U./kg B.W.) during insulin tolerance testing [ Arum *et al.*, 2009; Bonkowski *et al.*, 2006].

### Caloric restriction

Mice were 4–8 months of age at inception of restriction.

The amount of food allotted each cage of mice designated for caloric restriction was determined based on (weekly calculated) *ad libitum* food consumption for entire cages of gender-, genotype-, and birth date-matched controls; these values were averaged over the number of cages within each such group. Two hundred grams of the above-described food was placed in each A.L. cage-hopper on a weekly basis. After six days of food consumption, the remaining food was weighed on a Scout Pro Balance (Ohaus Co., Pine Brook, NJ) calibrated to weight standards on a monthly basis; food consumption values were calculated as follows: {(200 g.) – [food remaining after six days (in g.)]}/six (days)/number of subjects in cage.

**Figure 1.**
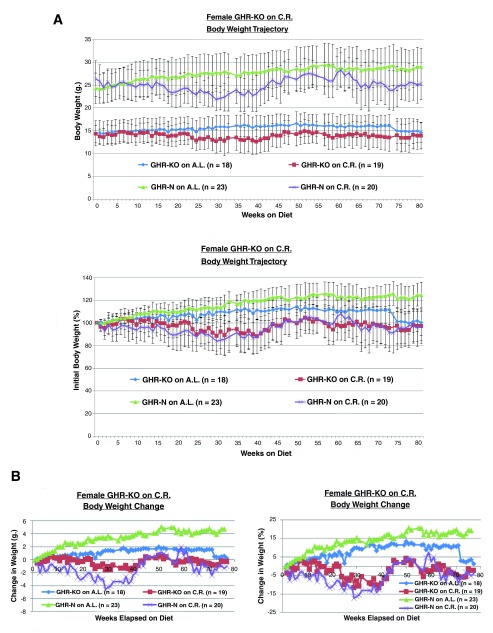
Effects of genotype and diet on body weight. **A.** 30% caloric restriction represses body weight gain (absolute or normalized-to-initial) in female GHR-KO mice and their littermate controls. **B.** 30% caloric restriction reins change in body weight (absolute or normalized-to-initial) in GHR-KO females and their littermate controls.

As a protection against dissimilar food consumption within CR cages, part of the food was broken into pieces small-enough to pass through the hopper-grate (but not crumbs). Our observations confirmed that this method allowed every restricted mouse to feed *ad libitum* during the initial surge of food consumption. Considering the valid concerns related to differential restriction resulting from a dominant cage-mate consuming more than their fair proportion, we paid particular attention to any individual mouse weight loss and health ( *e.g.* fight wounds indicative of physical conflicts with a cage-mate) throughout our studies. It is also worth noting that our chosen level of restriction (30%) is moderate compared to the 40% level that causes considerable concerns [ Liao *et al.*, 2010; Mattson, 2010]. This moderate level does not lead to an extinguishment of food supply after the initial gorge (thus, even subordinate mice have ample, albeit possibly delayed, access to food) and does not result in substantial weight loss for any sub-cohort of animal-subjects within our stocks ( Figure 1A and B).

Of relevance to obviating unintended interactions between experimental factors, which might produce confusing or obfuscating variation within the data, mice were housed in genotype-, age-, and diet-specific cages.

### Weekly body weight determinations and food consumption measurements

Mice were weighed weekly on a Scout Pro Balance (Ohaus Co., Pine Brook, NJ) that was calibrated to weight standards on a monthly basis. All mice were weighed in the late afternoon, approximately 20 hours after the restricted mice had been fed.

### Age-grade classification

Mice were young-adults in all experiments except for the indirect calorimetry trials involving CR, the spontaneous locomotor activity experiments and the behavior (anxiety & memory) experiments, where mice had to be middle-aged in order to address gerontological queries.

Age-staging was based on a combination of 1) quantitative extrapolation from prior stock-specific survivorship data [ Bonkowski *et al.*, 2006], 2) presence/appearance of aging-associated wizening (as represented quantitatively by declining body weight), and 3) spontaneous, testing-independent, (and presumably) aging-resultant mortality. Thus, young-adulthood is marked by at least 90% of reproductively competent negative control subjects being alive; middle-age is the period between when approximately 90% of the control subjects are still alive and median survivorship; old-age is the period between median survivorship and when approx. 10% of the subjects are alive; and oldest-old age is designated as the period when ≤ 10% of the controls remain.

### Blood glucose regulatory assessments

All animals underwent home-cage assessments of gross health ( Supplemental Table I) and any animal exhibiting questionable health by these criteria, or which was aberrantly hypoglycemic at the inception of a test, was excluded from the testing and/or data analysis. In addition, all animals were given at-least two weeks of recuperation in-between tests.

Glucose tolerance testing [ *ad libitum (A.L.)*-fed or fasted]

For A.L.-fed tests, animals had access to food for at least 16 hours before the test. For fasted tests, animals were fasted for 16 hours, although CR animals were A.L.-fed the day before the 16-hour fast commenced. Thirty minutes prior to beginning the test, each animal was weighed, had a small nick placed at the tip of its tail with a razor, and re-housed without access to food. After 30 minutes to recover from the handling stress of the weighing and tail-nicking, blood glucose concentration was assessed in each animal. Blood was obtained by applying a gentle pressure to the tail-tip, with a blood glucose monitoring system (glucometer and testing strips) (OneTouch Ultra 2, Lifescan, Inc., Milpitas, CA). Without releasing the grasp on the animal, it was manually repositioned to a nearly supine pose, and injected inter-peritoneally with 2 g. D-(+)-glucose (Sigma-Aldrich Co., St. Louis, MO) per kg of body weight. [The powdered glucose was dissolved in 0.9% sodium chloride (Sigma-Aldrich Co., St. Louis, MO)]. Subsequent blood glucose measurements were at 10, 20, 30, 40, 50, 60, 75, 90, and 120 minutes after the injection. Animals were given A.L. access to food immediately after completion of the test.

Insulin tolerance testing

Animals had access to food for at least 16 hours before the test. Animals were prepared for injection as described for glucose tolerance testing (above). Animals were injected inter-peritoneally with 0.75 U.S.P.U. of porcine insulin (Sigma-Aldrich Co., St. Louis, MO) per kg of body weight. (The lyophilized insulin was dissolved in 0.9% sodium chloride). Subsequent blood glucose measurements were at 10, 20, 30, 40, 50, 60, 75, 90, and 120 minutes after the injection. Animals were given A.L. access to food immediately after completion of testing.

Pyruvate conversion testing

Animals were fasted for 16 hours and CR animals were A.L.-fed the day before the fast commenced. Animals were prepared for injection as described for glucose tolerance testing (above). Animals were injected inter-peritoneally with 2 g of sodium pyruvic acid (Sigma-Aldrich Co., St. Louis, MO) per kg of body weight. (The lyophilized sodium pyruvate was dissolved in 0.9% sodium chloride). Subsequent blood glucose measurements were at 15, 30, 45, 60, and 120 minutes after the injection. Animals were given A.L. access to food immediately after completion of testing.

Non-stimulated blood glucose comparisons [ *ad libitum* (A.L.)-fed or fasted]

Un-stimulated blood glucose values were obtained from young-adult mice at the beginnings of A.L.-fed and fasted glucose tolerance tests, drawn from a small nick at the tip of the tail and measured with a blood glucose monitoring system (glucometer and testing strips) (OneTouch Ultra 2, Lifescan, Inc., Milpitas, CA). A.L.-fed blood glucose values were collected after an overnight (~16 hrs.) period of A.L. feeding for all subjects; fasted blood glucose values were gathered after equivalent overnight fasting (~16 hrs.) for all subjects.

A.L.-fed blood glucose values recorded immediately preceding a sacrifice and tissue harvesting from middle-aged mice were consistent with the results obtained as above. As a control against inferences drawn from the possible effects of short-term fasting, CR mice were noted to have stomachs freighted with foodstuff upon the sacrifice that followed the blood glucose assessment.

### A.L.-fed and fasted indirect calorimetry

Indirect calorimetry was conducted as previously described by Westbrook *et al.*, (2009) (Accuscan Instruments, Inc., Columbus, OH). Acclimation day testing, A.L.-fed day testing and fasted day testing were all conducted in one longitudinal stretch. Data were normalized per unit of lean body weight [ Butler & Kozak, 2010] as determined by fat depot sub-dissection [ Berryman *et al.*, 2010; Muzumdar *et al.*, 2008]. The values at the 17:00 hour were excluded from the statistical analyses, as this time was used for maintenance activities ( *e.g.* removal of food, weighing of remaining food, and weighing of mice) during longitudinal testing paradigm. Parameters assessed are annotated in Supplemental Table II a.

### A.L.-fed and fasted spontaneous locomotor activity

Spontaneous locomotion was assessed using the same equipment and the indirect calorimetry procedure described above (Accuscan Instruments, Inc., Columbus, OH). The values measured at the 17:00 hour were excluded from the statistical analyses, as this time was used for maintenance activities ( *e.g.* removal of food, weighing of remaining food, and weighing of mice) during longitudinal testing paradigm. Parameters assessed are annotated in Supplemental Table II b.

### Complete blood cell (C.B.C.) count analysis of hematopoietic cell parameters

Blood cell counting was accomplished using a VetScan HM2 Hematology System (Abaxis, Union City, CA) and ≥ 25 μL of whole blood [collected in EDTA-coated Microvette 100 μL capillary tubes (Sarstedt AG & Co., Nümbrecht, Germany)] drawn from *ad libitum*-fed subjects. The following 18 parameters were assessed: concentration of leukocytes/white blood cells (W.B.C.), concentration of lymphocytes (LYM), concentration of monocytes (MON), concentration of granulocytes (GRA), proportion of leukocytes that are lymphocytes (LYM%), proportion of leukocytes that are monocytes (MON%), proportion of leukocytes that are granulocytes (GRA%), concentration of erythrocytes/red blood cells (R.B.C.), concentration of hemoglobin (g/dL) (HGB), hematocrit (%) (HCT), mean (erythrocytic) cell volume (M.C.V.), mean corpuscular hemoglobin (pg.) (M.C.H.), mean corpuscular hemoglobin concentration (g/dL) (M.C.H.C.), red cell (erythrocytic) distribution width (%) (R.D.W.), concentration of thrombocytes/platelet cells (PLT), mean platelet volume (M.P.V.), plateletocrit/platelet hematocrit (%) (PCT), platelet distribution width (%) (P.D.W.).

### Insulin biochemical assays

Plasma insulin was measured with the multiplexed Mouse Endocrine Lincoplex ELISA kit (LINCO Research, St. Charles, MO).

### One-, two-, and five-minutes open-field chamber anxiety/exploratory inclination assay

All animals underwent home-cage assessments of gross health, locomotor ability and activity ( Supplemental Table I). Animals exhibiting questionable health based on these criteria were excluded from the testing.

During the light-phase of their day, the mice were individually placed in the center of a lid-less, opaque, white, 44 × 44 × 40 cm. (length × width × height) polymer box with the floor divided into 16 11 × 11 cm ^2^. The number of squares entered within the allotted time was noted per mouse per trial; as the experiment is contingent upon the novelty of the aberrant context, each subject was only tested once. The methods used derived from standard methodologies previously used “to analyze general activity and exploratory drive” [ Crawley, 2007; Selman *et al.*, 2009].

### Open-field chamber proximal long-term memory assay

All animals underwent home-cage assessments of gross health, and locomotor ability & activity ( Supplemental Table I). Animals exhibiting questionable health based on these criteria were excluded from the testing.

During the light-phase of their day, mice were individually placed in the center of a lid-less, opaque, white, 44 × 44 × 40 cm (length × width × height) polymer box with the floor divided into 16 11 × 11 cm ^2^. The number of squares entered within one minute was noted per mouse per trial; 24 hours after the initial evaluation (acquisition),the mice were re-tested (retention). Memory index values were calculated per mouse as follows: (Retention activity/Acquisition activity); these reflect the degree to which the subject remembered the context presented 24 hours prior (with enhanced memory putatively resulting in more movement due to less anxiety). Final location scores evaluate the ultimate (after the 60-second testing interval) position of a mouse on the retention day, with a more-ensconced placement being indicative of greater anxiety (and, thus, worse memory of prior context) than a more-exposed positioning. The methods derived from standard methodologies used by other investigators [ Crawley, 2007].

### Data presentation and statistical analysis

Graphs were generated with Excel (Microsoft, Redmond, WA) and IrfanView Image Viewer (Irfan Skiljan, Wiener Neustadt, Austria; http://www.irfanview.com/). The measures of central tendency are arithmetic means, and all depictions of variation (error bars) represent the standard deviations (S.D.) [ Glantz, 2002].

Pre- *hoc* statistical measures

In brief, experimental design approaches were taken to maximize robustness while lessening the potential need to increase sample size; utilizing 1) an *a priori* specification of a limited number of well-defined hypotheses, 2) refinement of experimental techniques, and 3) grouping of animals so that the effect of unit variability on the treatment was minimized.

Post- *hoc* statistical analysis

Levene’s tests (to investigate scedasticity) and Kolmogorov-Smirnov tests (to determine deviations from Gaussian distribution) were conducted to guide the choice of statistical algorithms for analysis of differences amongst groups. The combined parameters of effect size and Type 1 error probability were considered when determining phenomena meriting presentation and discussion.

Most data were contrasted with unpaired, homoscedastic Student’s *t*-test, Analysis of Variance, or Analysis of Variance for Repeated Measures (ANOVA or ANOVA-R.M., resp.), as appropriate; followed by the Tukey’s Honestly Significant Difference (H.S.D.) or the Dunnett’s *t*-test post- *hoc* tests for multiple pairwise comparisons, as appropriate.

For repeatedly measured blood glucose regulatory assessments, the *p*-value for a given pairwise comparison at a given time-point represents the result of testing all of the time-points, up-to-and-including that time-point, within the repeated measures analysis; this permits testing whether both groups have experienced similar excursions in blood glucose (the null hypothesis) relative to their initial values and with consideration of all intermediate values. This mode of analysis poses more discrete and descriptive inquiries than analyzing the area under respective curves or utilizing isolated, independent blood glucose values/percentages at lone time-points. The data that are normalized to initial blood glucose values were used for the precise, time-point-specific *p*-values reported, yet the inferences of differences amongst groups do not depend on the use of these normalized data. For a particular pairwise comparison within a particular assay, the *p*-value reported in the text is the most conservative ( *i.e.* highest) sub-0.05 *p*-value from the series of repeated measures analyses.

In instance of considerable variation in data confounding inferences, the data outside of 1 S.D. might have been equilaterally excluded from the data used for statistical analysis.

Statistical comparisons were conducted with PSPP for Windows (Free Software Foundation, Inc., http://www.gnu.org/software/pspp/get.html).

## Results

Probing parameters pursuant to proliferation

A 30% CR resulted in the standard body weight (B.W.) gain attenuation, whether represented in absolute grams or in percentage-of-initial ( Figure 1A) or in body weight change in grams or in percentage-of-initial ( Figure 1B).

When scrutinizing proliferation on a cellular level, hematocytometric analyses of various blood cell parameters ( *e.g.* erythrocytes, leukocytes, and platelets) in late-middle-aged (~25 months-of-age) revealed no effect of CR on either GHR-N mice or GHR-KO mice ( Table 1).

**Table 1.** Complete blood cell analysis reveals effects of *Ghr/bp* gene disruption, but not of caloric restriction.

Group	Weight (g.)	Blood Glucose (mg/dL)	WBC (10^9/L)	LYM (10^9/L)	MON (10^9/L)	GRA (10^9/L)	LY% (%)	MO% (%)	GR% (%)	RBC (10^12/L)	HGB (g/dL)	HCT (%)	MCV (fL)	MCH (pg)	MCHC (g/dL)	RDWc (%)	PLT (10^9/L)	PCT (%)	MPV (fL)	PDWc (%)
**GHR-KO on** **A.L. (n = 15)**	*15.9333*	*110.6*	*9.5613*	*8.8153*	*0.3447*	*0.4087*	*92.3333*	*3.4067*	*4.4733*	*7.986*	*10.72*	*38.0713*	*48.2*	*13.42*	*27.9133*	*19.4333*	*169*	*0.1093*	*6.5533*	*33.8067*
**GHR-KO on** **C.R. (n = 18)**	*15.1267*	*74*	*6.3639*	*5.1517*	*0.1883*	*1.0294*	*81.5056*	*2.6778*	*15.9333*	*7.6256*	*10.3056*	*37.1589*	*48.7222*	*13.4389*	*27.5833*	*19.5778*	*169.7222*	*0.1133*	*6.6111*	*33.8389*
**GHR-N on** **A.L. (n = 13)**	*28.2846*	*123.1538*	*8.6477*	*7.0115*	*0.3231*	*1.3138*	*80.1*	*3.8154*	*16.0769*	*10.4108*	*15.0846*	*49.8562*	*47.8462*	*14.4769*	*30.2615*	*18.2538*	*210.3077*	*0.1462*	*6.6923*	*33.2769*
**GHR-N on** **C.R. (n = 7)**	*27.6857*	*93.2857*	*6.8171*	*5.0771*	*0.3071*	*1.4357*	*75.9*	*4.3714*	*19.7286*	*9.6171*	*13.6571*	*46.1057*	*48*	*14.1857*	*29.6143*	*18.3857*	*209.1429*	*0.1529*	*7.4429*	*36.3143*
**Comparison**	**Weight** **(g.)**	**Blood** **Glucose** **(mg/dL)**	**WBC** **(10^9/L)**	**LYM** **(10^9/L)**	**MON** **(10^9/L)**	**GRA** **(10^9/L)**	**LY%** **(%)**	**MO%** **(%)**	**GR%** **(%)**	**RBC** **(10^12/L)**	**HGB** **(g/dL)**	**HCT** **(%)**	**MCV** **(fL)**	**MCH** **(pg)**	**MCHC** **(g/dL)**	**RDWc** **(%)**	**PLT** **(10^9/L)**	**PCT** **(%)**	**MPV** **(fL)**	**PDWc** **(%)**
**N on A.L.** *vs.* **KO on A.L.**	**0.0001**	**0.0504**	0.8781	0.6014	0.3778	**0.0316**	**0.04**	0.149	**0.0791**	**0.0001**	**0.0001**	**0.0001**	0.3932	**0.0001**	**0.0001**	**0.0067**	0.1624	**0.0766**	**0.0962**	0.2792
**N on A.L.** *vs.* **N on C.R.**	0.8328	0.7483	0.491	0.3614	0.8382	0.641	0.4016	0.8645	0.399	0.7149	0.6556	0.6557	0.7437	0.5829	0.8362	0.6758	0.9446	0.9004	0.5117	0.3194
**N on A.L.** *vs.* **KO on C.R.**	**0.0001**	**0.0206**	0.978	0.513	0.475	**0.0561**	**0.077**	0.2159	0.1407	**0.0001**	**0.0001**	**0.0001**	0.1039	**0.0001**	**0.0001**	**0.0006**	0.2506	0.1568	0.126	0.3383
**KO on A.L.** *vs.* **KO on C.R.**	0.6774	0.8026	0.8524	0.9033	0.8787	0.8463	0.8021	0.865	0.8167	0.996	0.8345	0.8294	0.6563	0.4264	0.7119	0.6605	0.8007	0.7092	0.9049	0.9199

Blood glucose homeostatic regulation experiments

**Figure 2.**
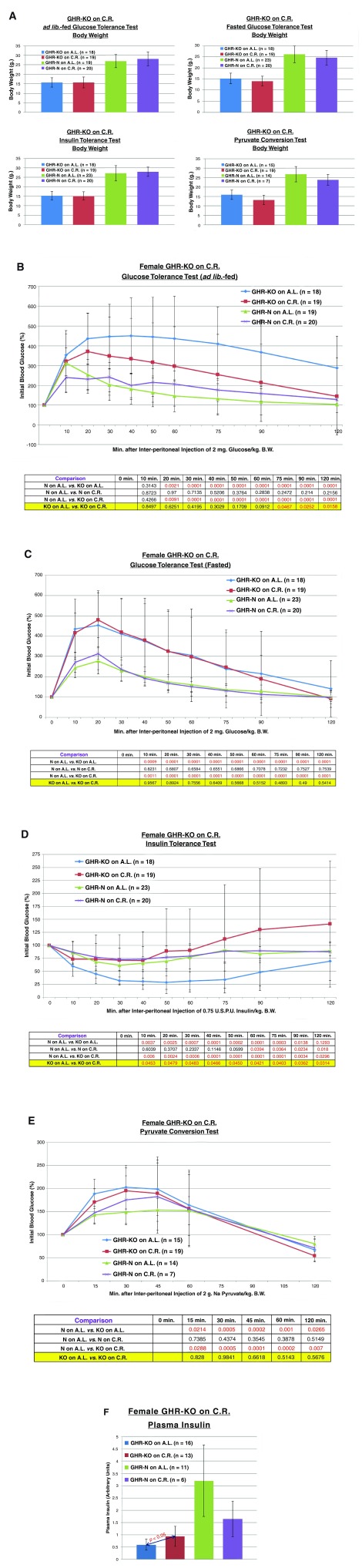
Endocrinological assessment of effect of 30% caloric restriction on GHR-KO mouse. **A.** 30% caloric restriction does not affect body weight immediately preceding a tolerance or conversion test for GHR-N or GHR-KO females. **B.** 30% caloric restriction partially corrects the glucose intolerance of female GHR-KO mice under A.L.-fed conditions (including repeated-measures statistical analysis table). **C.** 30% caloric restriction does not affect the glucose intolerance of GHR-KO females under fasted conditions (including repeated-measures statistical analysis table). **D.** 30% caloric restriction corrects the enhanced insulin sensitivity of female GHR-KO mice (including repeated-measures statistical analysis table). **E.** 30% caloric restriction does not significantly alter the heightened *de novo* hepatic glucose production of GHR-KO females (including repeated-measures statistical analysis table). **F.** 30% caloric restriction increases plasma insulin in female GHR-KO mice.

Importantly, there was no effect of the 30% CR diet on B.W. measured immediately preceding the testing for any of the blood glucose homeostasis regulation assays ( Figure 2A).

In relation to our hypothesis, CR increased A.L.-fed glucose incorporation in fed female GHR-KO mice during glucose tolerance testing [( *p* = 0.0467), ( Figure 2B)], but had no effect in fasted GHR-KO mice ( Figure 2C). As for the insulin tolerance tests, CR attenuated the sensitivity of GHR-KO females to 0.75 U.S.P.U./kg B.W. of insulin [( *p* = 0.0483), ( Figure 2D)]. CR did not alter the pyruvate conversion potential in female GHR-KO mice ( Figure 2E). Additionally, CR increased the plasma insulin content in GHR-KO mice [( *p* < 0.05, ( Figure 2F)].

Therefore, our data show additive or synergistic effects of CR with the GHR-KO gene disruption on blood glucose homeostasis.

Indirect measures of metabolism

Measurements estimating the general rate of metabolic processes have long been correlated with ultimate survivorship, and have been proffered as sufficient to explain the rate of senescence [ Rubner, 1908]. Whether the mechanisms by which CR retards senescence include alterations (particularly, decreases) in metabolism has been an active research hypothesis for some time [ Ramsey *et al.*, 2000].

Indirect (gas exchange) calorimetric measurements of metabolism have been reported to be increased [ Westbrook *et al.*, 2009], as well as decreased [ Carrillo & Flouris, 2011; Mookerjee *et al.*, 2010], in animals with extended longevity. Identifying metabolic phenotypes that transcend one particular genetic background or mode of delaying and/or decelerating aging would be important for proposing or testing mechanisms of extended lifespan and healthspan.

During our analyses of oxygen consumption (VO _2_), respiratory quotient (R.Q.)/respiratory exchange ratio (R.E.R.), heat production (Calories/hr), and energy expenditure (E.E.) in A.L.-fed and fasted female GHR-KO mice on CR, no genotype- or diet-based differences were detected for food consumption, changes in body weight induced by either acclimation or fasting, or thermogenesis (as crudely measured with an ambient thermometer in each chamber) while the subjects were in the indirect calorimetry chambers from the acclimation day through the A.L.-fed day to the fasted day ( Table 2).

**Table 2.** Assessments within indirect calorimetry/spontaneous activity chambers detail lack of genotype- or diet-based differences for food consumption, body weight, or thermogenesis.

Group	Body Wt. (g.) on Day After.....			Food Remaining (g.) on Day After…..			Chamber Temp. (°C) on Day…..			
	Acclimation	A.L.-feeding	Fasting	Acclimation	A.L.-feeding	Fasting	Before Acclimation	Before A.L. -feeding	Before Fasting	After Fasting
**GHR-KO on A.L.** **(n = 15)**	*15.7133*	*15.48*	*14.7067*	*22.0667*	*19.3733*		*23.95*	*24.0111*	*24.2333*	*23.9778*
**GHR-KO on C.R.** **(n = 19)**	*14.3737*	*14.1632*	*13.4526*	*21.7421*	*19.1053*		*23.8*	*24.0923*	*24.0308*	*23.9083*
**GHR-N on A.L.** **(n = 13)**	*27.7*	*27.2923*	*26.0308*	*21.7077*	*18.3167*		*23.7111*	*24.0333*	*24.0556*	*24.3222*
**GHR-N on C.R.** **(n = 7)**	*28.4429*	*28.4857*	*26.7*	*18.9*	*14.0286*		*23.6333*	*23.95*	*23.8833*	*24.1667*

*n.b.*: 25 g. of food placed in each chamber at beginning of acclimation day

CR did not affect these A.L.-fed indirect calorimetry-based measures of metabolism effects of female littermate controls, nor that of female GHR-KO mice ( Table 3).

**Table 3.** Caloric restriction did not affect A.L.-fed indirect calorimetry-based measures of metabolism.

Group	VO _2_	VCO _2_	R.Q. (R.E.R.)	Heat Prod.	Energy Exp.
**GHR-KO on A.L. (n = 15)**	*91.6681*	*56.0253*	*0.6533*	*412.90187916116*	*1772.53037940749*
**GHR-KO on C.R.** **(n = 17)**	*94.9652*	*59.5818*	*0.7117*	*375.235233147689*	*1843.84039232303*
**GHR-N on A.L. (n = 12)**	*46.0284*	*39.6722*	*0.8636*	*385.530070007391*	*948.172708705627*
**GHR-N on C.R. (n = 7)**	*54.6331*	*51.8225*	*0.8973*	*434.147*	*1149.1911042591*
Comparison	VO _2_	VCO _2_	R.Q. (R.E.R.)	Heat Prod.	Energy Exp.
**N on A.L.** *vs.* **KO on A.L.**	**0.0001**	**0.0001**	**0.0001**	0.194	**0.0001**
**N on A.L.** *vs.* **N on C.R.**	0.0262	**0.0001**	0.0601	0.0966	**0.0027**
**N on A.L.** *vs.* **KO on C.R.**	**0.0001**	**0.0001**	**0.0001**	0.6373	**0.0001**
**KO on A.L.** *vs.* **KO on C.R.**	0.4652	0.0139	**0.0026**	0.0248	0.3517

Spontaneous locomotor activity late in life, as it can serve as a measure of the multi-factorial syndrome of frailty [ Walston *et al.*, 2006] ( *i.e.* frail mice would presumably be disinclined to move, or cover less area when trying) is often used as a behavioral marker of delayed and/or decelerated senescence [ Ingram, 2000; Manini, 2010; Minor *et al.*, 2011; Neff *et al.*, 2013; Wilkinson *et al.*, 2012; Zhang *et al.*, 2014].

Similarly, CR had no germane effect on the spontaneous locomotion of female littermate control mice, or female GHR-KO mice ( Table 4).

**Table 4.** Caloric restriction did not affect A.L.-fed measures of spontaneous locomotion.

Group	Total Distance	Hor. Activity Ct.	Amb. Activity Ct.	Rest Time	Rest Episode Ct.	Mvmt. Time	Mvmt. Episode Ct.	Stereo. Time	Stereo. Episode Ct.	Stereo. Activity Ct.	Vert. Episode Ct.	Vert. Activity Ct.	Vert. Activity Time	Loco. Clock. Revs.	Loco. Counter. Revs.
**GHR-KO on A.L. (n = 15)**	*56.3851*	*192.609661835749*	*163.115458937198*	*556.624415458937*	*38.1217*	*43.3094*	*37.3903*	*7.9026*	*12.5841*	*29.4942*	*3.8304*	*6.8155*	*2.5093*	*0.2681*	*0.229*
**GHR-KO on C.R. (n = 17)**	*64.2217*	*216.63409610984*	*182.195499618612*	*553.747614797864*	*41.5477*	*46.1707*	*40.8561*	*8.9517*	*14.3607*	*34.4386*	*7.1078*	*6.7298*	*15.9952*	*0.3376*	*0.2122*
**GHR-N on A.L. (n = 12)**	*56.9752*	*204.620531400966*	*171.050362318841*	*554.467710144928*	*41.1762*	*45.4566*	*40.4714*	*9.177*	*13.7315*	*33.5702*	*8.971*	*25.8674*	*11.2301*	*0.2286*	*0.2367*
**GHR-N on C.R. (n = 7)**	*68.7457*	*231.376811594203*	*191.912*	*550.773944099379*	*42.2298*	*49.1525*	*41.528*	*10.6875*	*15.7495*	*39.4648*	*9.2143*	*25.2443*	*9.9293*	*0.2557*	*0.3302*
Comparison	Total Distance	Hor. Activity Ct.	Amb. Activity Ct.	Rest Time	Rest Episode Ct.	Mvmt. Time	Mvmt. Episode Ct.	Stereo. Time	Stereo. Episode Ct.	Stereo. Activity Ct.	Vert. Episode Ct.	Vert. Activity Ct.	Vert. Activity Time	Loco. Clock. Revs.	Loco. Counter. Revs.
**N on A.L.** *vs.* **KO on A.L.**	0.9371	0.5322	0.6443	0.5747	0.1661	0.576	0.1659	0.0299	0.2379	0.0867				0.4116	0.8377
**N on A.L.** *vs.* **N on C.R.**	0.1801	0.2782	0.3389	0.4354	0.724	0.4345	0.7255	0.0603	0.1187	0.0688	0.8449	0.8843	0.4361	0.5075	0.094
**N on A.L.** *vs.* **KO on C.R.**	0.3753	0.5288	0.5084	0.837	0.8632	0.8381	0.8595	0.7026	0.5453	0.7347				0.1661	0.3991
**KO on A.L.** *vs.* **KO on C.R.**	0.3584	0.211	0.2654	0.4124	0.0766	0.4144	0.0756	0.0496	0.0743	0.0396	**0.0002**	0.923	**0.0001**	0.3678	0.6131

Cognitive assessments

The retention of cognitive capability (in particular, memory function) into middle-age and beyond in the GHR-KO mice further supports that the effects of the *Ghr/bp* disruption extend beyond increasing survivorship, to include ameliorating senescence and its resultant functional decrements [ Bartke, 2005; Kinney *et al.*, 2001a; Kinney *et al.*, 2001b; Kinney-Forshee *et al.*, 2004; O. Arum & A. Bartke, (unpublished data)]. With noteworthy exceptions, which are partly due to varied methodologies of caloric restriction or cognitive assessment amongst scientists, CR is broadly considered to be beneficial for retarding aging-resultant cognitive decline [ Arslan-Ergul *et al.*, 2013; Joseph *et al.*, 2009].

**Figure 3.**
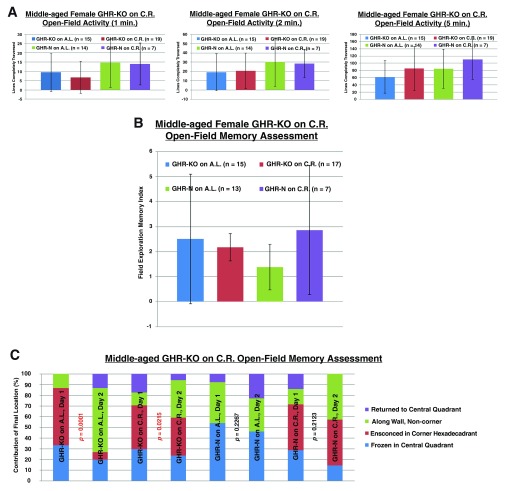
Failure of 30% caloric restriction to influence cognition. **A.** Neither *Ghr/bp* disruption nor 30% caloric restriction alters anxiety-betraying activity in an open field for female mice. **B.** 30% caloric restriction does not change the (memory index) performance of female GHR-KO mice in the open-field paradigm. **C.** 30% caloric restriction does not change the (final location) performance of female GHR-KO mice in the open-field paradigm.

Regarding the cognitive assessments, CR had no effect on the anxiety of either littermate control or GHR-KO females ( Figure 3A). The memory index results derived from open-field activity ( Figure 3B) did not support the initial hypothesis that the greater insulin sensitivity of GHR-KO mice precludes their full benefits from CR (as the GHR-KO mice on A.L. and the GHR-KO mice on CR, which have differing insulin responsiveness, did not differ in memory performance). Similar inferences were concluded for the open-field activity-based memory tests regarding the final location of the mice ( Figure 3C).

Experimental data showing the effect(s) of growth hormone receptor/binding protein (Ghr/bp) gene disruption and/or caloric restriction on the various outcomes Data showing the effects of either the senescence-retarding Ghr/bp mutation, the senescence-retarding 30% caloric restriction, or both factors on the following (groups of) traits: 1) body weight, 2) complete blood cell content, 3) food consumption, 4) ad libitum-fed glucose tolerance, 5) fasted glucose tolerance, 6) 0.75 U.S.P.U. insulin tolerance, 7) 0.3 U.S.P.U. insulin tolerance, 8) pyruvate conversion, 9) open-field activity, 10) open-field memory, 11) various characteristics peripheral to the indirect calorimetry and spontaneous activity chambers-based experiments, 12) ad libitum-fed indirect calorimetry and spontaneous activity, and 13) fasted indirect calorimetry and spontaneous activity.

## Discussion

The initial aim of this study was to investigate if the very limited response of the GHR-KO mouse to a 30% CR diet in terms of longevity [ Bonkowski *et al.*, 2006] is related to the inability of these mutants to respond to a 30% CR diet with regards to insulin sensitivity [ Bonkowski *et al.*, 2006]. This was based on the hypothesis that it is the maximization of the response to CR in the insulin sensitivity test that acts as a “ceiling/floor” effect limiting the survivorship response to CR [ Bonkowski *et al.*, 2006]. Our insulin sensitivity results in GHR-KO mice on 30% CR differed from those obtained in a previous study showing that caloric restriction promotes euglycemia in GHR-KO mice ( Figure 2C). These differences might have been due to the difference in ages of the animals {12 months in [ Bonkowski *et al.*, 2006] *vs.* 8–13 months in the present report}, or different durations of CR (10 or 12 months *vs.* 4–6 months, respectively). Those *caveats emptor* notwithstanding, that blood insulin content is increased by CR in GHR-KO mice ( Figure 2E) dovetails with the improved performance in glucose bolus assimilation ( Figure 2A), decreased insulin sensitivity ( Figure 2C), and (statistically indistinguishable) decreased gluconeogenic capability ( Figure 2D) of GHR-KO mice on CR relative to their A.L. counterparts. Moreover, data from macromolecular analysis of insulin signaling in GHR-KO mice on CR, including decreased insulin receptor (INSR) and thymoma viral proto-oncogene 1/protein kinase b (AKT1/PKB) concentrations in the skeletal musculature of GHR-KO’s on CR, and decreased phosphatidylinositol-4,5-bisphosphate 3-kinase (PI3K) subunits content in the livers of GHR-KO’s on CR (relative to GHR-KO’s on AL), also corroborate and portend decreased insulin sensitivity in GHR-KO mice on CR [ Bonkowski *et al.*, 2009]. Additionally, it is worth noting that a tight regulation of euglycemia would be more consistent with health and survival than a predilection for hypoglycemia [ Tan & Flanagan, 2013], thus “improving health”, as CR has been broadly documented as doing, might mean preventing the innate endocrinological/metabolic derangements that are merely coincident with the longevity of the GHR-KO mouse. Finally, to the best of our knowledge, published reports on CR-mediated induction of insulin sensitivity ( *vis-à-vis* increased blood glucose assimilation dynamics) using insulin tolerance tests or hyperinsulinemic-euglycemic clamping assays on healthy mice are either lacking or are not consistently reproduced. This is an important limitation, and *caveat emptor*, given that mutant mice with abnormal growth and adult body composition have been documented to have insulin tolerance testing results in disagreement with the molecular biology-based assumptions of their insulin sensitivity [ Boparai *et al.*, 2010].

We also investigated the effects of CR on the performance of GHR-KO mice in other gerontologically associated measures. We discovered that CR did not alter the metabolism or spontaneous activity of GHR-KO mice and also revealed that CR has no effect on the anxiety or memory function of GHR-KO mice. This documentation of lacking amenability of GHR-KO mice to effects of CR further underscore a seeming epistasis of the genetic effect of *Ghr/bp* disruption to the environmental effect of dietary restriction.

In summary, our results question the notion of maximized insulin sensitivity obviating further lifespan increase in GHR-KO mice. Future studies aimed at elucidating concordant physiological, and ultimately (macro)molecular, underpinnings of disparate instances of longevity would benefit from heeding analyses that reduce or eliminate the likelihood of suspected mechanisms.

## Data availability

Dataset 1. Experimental data showing the effect(s) of *growth hormone* (GH) *receptor* (GHR) */binding protein (Ghr/bp)* gene disruption and/or caloric restriction on the various outcomes, http://dx.doi.org/10.5256/f1000research.5378.d37530 [ Arum *et al.*, 2014].

## Figures and Tables

**Supplemental Table I.** Home-Cage assessment rubric. Characteristics of interest in home-cage assessments are succinctly detailed.

Condition	Rating	Description
Initial Posture	1	Sitting or standing normally, rearing or asleep
	2	Crouching over or lying low
	3	Slight sway while in standing position
	4	Excessive sway or head bobbing in standing position
	5	Flattened, limbs may be spread out
	6	Lying on side, limbs in air
Salivation	1	None
	2	Slight
	3	Severe
Lacrimation	1	None
	2	Slight
	3	Severe
Fur	1	Normal, silky and smooth
	2	Pilo-erection
	3	Over groomed
Vocalization	0	No, spontaneous vocals
	1	Yes, spontaneous vocals

**Supplemental Table II a.** Legend for indirect calorimetry-based metabolic dependent variables.

**VO _2_**(Volume of oxygen consumed per hour, normalized to body weight.)
**VCO _2_** (Volume of carbon dioxide produced per hour, normalized to body weight.)
**Respiratory Quotient (Respiratory Exchange Ratio)** (VCO _2_/VO _2_)
**Heat Production** [((4.33 × VO _2_) + (0.67 × VCO _2_)) + (Wt.(kg.)) × (60 Min./Hr.)]
**Energy Expenditure**[VO _2_ × (364 + 113 × R.Q.)/22.4]

**Supplemental Table II b.** Legend for voluntary locomotion dependent variables.

**Total distance** [The total distance that the subject has traveled. For the purpose of this variable, the location of the subject is defined as the centroid (center of mass) of the subject.]
**Horizontal activity count**[A count of sensor changes (beam breaks).]
**Ambulatory activity count** (Number of beam breaks while animal is ambulating. Does not include stereotypy behavior.)
**Rest time** (The length of time that the subject spent at rest. A resting period is defined as a period of inactivity greater than or equal to 1 second.)
**Rest episode count** (The total number of resting periods. A resting period is defined as a period of inactivity greater than or equal to 1 second.)
**Movement time**(The length of time that the subject spent in activity. Activity is defined as a period in which ambulation or stereotypy occurred.)
**Movement episode count** (The total number of locomotor episodes. Episodes are separated by rest periods of at least 1 second.)
**Stereotypy time** [The total amount of time that stereotypic behavior is exhibited. A break in stereotypy of 1 second or more is required to separate one stereotypic episode from the next. If the animal breaks the same beam (or set of beams) repeatedly then the monitor considers that the animal is exhibiting stereotypy. This typically happens during grooming, head bobbing, *etc.*]
**Stereotypy episode count** [This corresponds to the number of times that stereotypic behavior was observed in the animal. A break in stereotypy of 1 second or more is required to separate one stereotypic episode from the next. If the animal breaks the same beam (or set of beams) repeatedly then the monitor considers that the animal is exhibiting stereotypy. This typically happens during grooming, head bobbing, *etc.*]
**Stereotypy activity count** [Number of beam breaks that occur during a period of stereotypic activity. If the animal breaks the same beam (or set of beams) repeatedly then the monitor considers that the animal is exhibiting stereotypy. This typically happens during grooming, head bobbing, *etc.*]
**Vertical episode count** (Each time the animal rears up, this is incremented by 1. The animal must go below the level of the vertical sensor for at least 1 second before the next rearing can be registered.)
**Vertical activity count** (Cumulative vertical beam breaks.)
**Vertical activity time** (When the animal rears up, this timer starts incrementing.)
**Locomotor clockwise revolutions** (Counts the number of clockwise revolutions that the subject travels in an open field.)
**Locomotor counter-clockwise revolutions** (Counts the number of counter- clockwise revolutions that the subject travels in an open field.)

## References

[ref-1] Arslan-Ergul A Ozdemir AT Adams MM: Aging, neurogenesis, and caloric restriction in different model organisms. *Aging Dis.* 2013;4(4):221–32. 23936746 PMC3733585

[ref-2] Arum O Bonkowski MS Rocha JS: The growth hormone receptor gene-disrupted mouse fails to respond to an intermittent fasting diet. *Aging Cell.* 2009;8(6):756–60. 10.1111/j.1474-9726.2009.00520.x 19747233 PMC2783987

[ref-3] Arum O Saleh JK Boparai RK: Experimental data showing the effect(s) of growth hormone receptor/binding protein (Ghr/bp) gene disruption and/or caloric restriction on the various outcomes. *F1000Research.* 2014 Data Source 10.12688/f1000research.5378.1 PMC4358413 25789159

[ref-4] Bartke A: Insulin resistance and cognitive aging in long-lived and short-lived mice. *J Gerontol A Biol Sci Med Sci.* 2005;60(1):133–4. 10.1093/gerona/60.1.133 15741298

[ref-5] Bartke A: Insulin and aging. *Cell Cycle.* 2008;7(21):3338–43. 10.4161/cc.7.21.7012 18948730

[ref-6] Bartke A Wright JC Mattison JA: Extending the lifespan of long-lived mice. *Nature.* 2001;414(6862):412. 10.1038/35106646 11719795

[ref-7] Berryman DE List EO Palmer AJ: Two-year body composition analyses of long-lived GHR null mice. *J Gerontol A Biol Sci Med Sci.* 2010;65(1):31–40. 10.1093/gerona/glp175 19901018 PMC2796884

[ref-8] Bonkowski MS Dominici FP Arum O: Disruption of growth hormone receptor prevents calorie restriction from improving insulin action and longevity. *PLoS One.* 2009;4(2):e4567. 10.1371/journal.pone.0004567 19234595 PMC2639640

[ref-9] Bonkowski MS Rocha JS Masternak MM: Targeted disruption of growth hormone receptor interferes with the beneficial actions of calorie restriction. *Proc Natl Acad Sci U S A.* 2006;103(20):7901–5. 10.1073/pnas.0600161103 16682650 PMC1458512

[ref-10] Boparai RK Arum O Khardori R: Glucose homeostasis and insulin sensitivity in growth hormone-transgenic mice: a cross-sectional analysis. *Biol Chem.* 2010;391(10):1149–1155. 10.1515/BC.2010.124 20707609 PMC4009680

[ref-11] Brown-Borg HM Borg KE Meliska CJ: Dwarf mice and the ageing process. *Nature.* 1996;384(6604):33. 10.1038/384033a0 8900272

[ref-12] Butler AA Kozak LP: A recurring problem with the analysis of energy expenditure in genetic models expressing lean and obese phenotypes. *Diabetes.* 2010;59(2):323–9. 10.2337/db09-1471 20103710 PMC2809965

[ref-13] Carrillo AE Flouris AD: Caloric restriction and longevity: effects of reduced body temperature. *Ageing Res Rev.* 2011;10(1):153–62. 10.1016/j.arr.2010.10.001 20969980

[ref-14] Conover CA Bale LK: Loss of pregnancy-associated plasma protein A extends lifespan in mice. *Aging Cell.* 2007;6(5):727–9. 10.1111/j.1474-9726.2007.00328.x 17681037

[ref-15] Conover CA Mason MA Levine JA: Metabolic consequences of pregnancy-associated plasma protein-A deficiency in mice: exploring possible relationship to the longevity phenotype. *J Endocrinol.* 2008;198(3):599–605. 10.1677/JOE-08-0179 18566100 PMC2593875

[ref-16] Coschigano KT Clemmons D Bellush LL: Assessment of growth parameters and life span of GHR/BP gene-disrupted mice. *Endocrinology.* 2000;141(7):2608–13. 10.1210/endo.141.7.7586 10875265

[ref-17] Coschigano KT Holland AN Riders ME: Deletion, but not antagonism, of the mouse growth hormone receptor results in severely decreased body weights, insulin, and insulin-like growth factor I levels and increased life span. *Endocrinology.* 2003;144(9):3799–810. 10.1210/en.2003-0374 12933651

[ref-18] Crawley JN: What’s Wrong With My Mouse? Behavioral Phenotyping of Transgenic and Knockout Mice, Second Edition. John Wiley & Sons, Inc. Hoboken, NJ.2007 Reference Source

[ref-19] Dominici FP Hauck S Argentino DP: Increased insulin sensitivity and upregulation of insulin receptor, insulin receptor substrate (IRS)-1 and IRS-2 in liver of Ames dwarf mice. *J Endocrinol.* 2002;173(1):81–94. 10.1677/joe.0.1730081 11927387

[ref-20] Glantz SA: Primer of Biostatistics, Fifth Edition. Chapter, Two: How to Summarize Data. The McGraw-Hill Companies, Inc. New York, NY.2002.

[ref-21] Ingram DK: Age-related decline in physical activity: generalization to nonhumans. *Med Sci Sports Exerc.* 2000;32(9):1623–9. 10.1097/00005768-200009000-00016 10994915

[ref-22] Joseph J Cole G Head E: Nutrition, brain aging, and neurodegeneration. *J Neurosci.* 2009;29(41):12795–801. 10.1523/JNEUROSCI.3520-09.2009 19828791 PMC6665319

[ref-23] Kenyon CJ: The genetics of ageing. *Nature.* 2010;464(7288):504–12. Erratum in: *Nature.*2010; 467(7315): 622. 10.1038/nature08980 20336132

[ref-24] Kinney BA Coschigano KT Kopchick JJ: Evidence that age-induced decline in memory retention is delayed in growth hormone resistant GH-R-KO (Laron) mice. *Physiol Behav.* 2001a;72(5):653–60. 10.1016/S0031-9384(01)00423-1 11336996

[ref-25] Kinney BA Meliska CJ Steger RW: Evidence that Ames dwarf mice age differently from their normal siblings in behavioral and learning and memory parameters. *Horm Behav.* 2001b;39(4):277–84. 10.1006/hbeh.2001.1654 11374913

[ref-26] Kinney-Forshee BA Kinney NE Steger RW: Could a deficiency in growth hormone signaling be beneficial to the aging brain? *Physiol Behav.* 2004;80(5):589–94. 10.1016/j.physbeh.2003.10.018 14984790

[ref-27] Lawler DF Larson BT Ballam JM: Diet restriction and ageing in the dog: major observations over two decades. *Br J Nutr.* 2008;99(4):793–805. 10.1017/S0007114507871686 18062831

[ref-28] Liao CY Rikke BA Johnson TE: Genetic variation in the murine lifespan response to dietary restriction: from life extension to life shortening. *Aging Cell.* 2010;9(1):92–5. 10.1111/j.1474-9726.2009.00533.x 19878144 PMC3476836

[ref-29] Liu JL Coschigano KT Robertson K: Disruption of growth hormone receptor gene causes diminished pancreatic islet size and increased insulin sensitivity in mice. *Am J Physiol Endocrinol Metab.* 2004;287(3):E405–13. 10.1152/ajpendo.00423.2003 15138153

[ref-30] Longo VD Finch CE: Evolutionary medicine: from dwarf model systems to healthy centenarians? *Science.* 2003;299(5611):1342–6. 10.1126/science.1077991 12610293

[ref-31] Manini TM: Energy expenditure and aging. *Ageing Res Rev.* 2010;9(1):1–11. 10.1016/j.arr.2009.08.002 19698803 PMC2818133

[ref-32] Masoro EJ: Subfield history: caloric restriction, slowing aging, and extending life. *Sci Aging Knowledge Environ.* 2003;2003(8):RE2. 10.1126/sageke.2003.8.re2 12844547

[ref-33] Masoro EJ: Overview of caloric restriction and ageing. *Mech Ageing Dev.* 2005;126(9):913–22. 10.1016/j.mad.2005.03.012 15885745

[ref-34] Mattison JA Roth GS Lane MA: Dietary restriction in aging nonhuman primates. *Interdiscip Top Gerontol.* 2007;35:137–58. 10.1159/000096560 17063037

[ref-35] Mattson MP: Genes and behavior interact to determine mortality in mice when food is scarce and competition fierce. *Aging Cell.* 2010;9(3):448–9; Discussion 450–2. 10.1111/j.1474-9726.2010.00561.x 20156203

[ref-36] Miller RA: “Dividends” from research on aging--can biogerontologists, at long last, find something useful to do? *J Gerontol A Biol Sci Med Sci.* 2009;64(2):157–60. 10.1093/gerona/gln062 19225032 PMC2655023

[ref-37] Miller RA Burke D Nadon N: Announcement: four-way cross mouse stocks: a new, genetically heterogeneous resource for aging research. *J Gerontol A Biol Sci Med Sci.* 1999;54(8):B358–60. 10.1093/gerona/54.8.B358 10496542

[ref-38] Minor RK Baur JA Gomes AP: SRT1720 improves survival and healthspan of obese mice. *Sci Rep.* 2011;1:70. Erratum in: *Sci Rep.*2011; 2013; 3: 1131. 10.1038/srep00070 22355589 PMC3216557

[ref-39] Mookerjee SA Divakaruni AS Jastroch M: Mitochondrial uncoupling and lifespan. *Mech Ageing Dev.* 2010;131(7–8):463–72. 10.1016/j.mad.2010.03.010 20363244 PMC2924931

[ref-40] Muzumdar R Allison DB Huffman DM: Visceral adipose tissue modulates mammalian longevity. *Aging Cell.* 2008;7(3):438–40. 10.1111/j.1474-9726.2008.00391.x 18363902 PMC2504027

[ref-41] Neff F Flores-Dominguez D Ryan DP: Rapamycin extends murine lifespan but has limited effects on aging. *J Clin Invest.* 2013;123(8):3272–91. 10.1172/JCI67674 23863708 PMC3726163

[ref-42] Olshansky SJ Perry D Miller RA: Pursuing the longevity dividend: scientific goals for an aging world. *Ann N Y Acad Sci.* 2007;1114:11–3. 10.1196/annals.1396.050 17986572

[ref-43] Piper MD Bartke A: Diet and aging. *Cell Metab.* 2008;8(2):99–104. 10.1016/j.cmet.2008.06.012 18680711

[ref-44] Ramsey JJ Harper ME Weindruch R: Restriction of energy intake, energy expenditure, and aging. *Free Radic Biol Med.* 2000;29(10):946–68. 10.1016/S0891-5849(00)00417-2 11084284

[ref-45] Rubner M: Das Problem der lebensdauer und seine beziehungen zu wachstum und erhahrung. *Munich: Oldenberg.* 1908 Reference Source

[ref-46] Selman C Lingard S Choudhury AI: Evidence for lifespan extension and delayed age-related biomarkers in insulin receptor substrate 1 null mice. *FASEB J.* 2008;22(3):807–18. 10.1096/fj.07-9261com 17928362

[ref-47] Selman C Tullet JM Wieser D: Ribosomal protein S6 kinase 1 signaling regulates mammalian life span. *Science.* 2009;326(5949):140–4. 10.1126/science.1177221 19797661 PMC4954603

[ref-48] Tan HK Flanagan D: The impact of hypoglycaemia on patients admitted to hospital with medical emergencies. *Diabet Med.* 2013;30(5):574–80. 10.1111/dme.12123 23323805

[ref-49] Walston J Hadley EC Ferrucci L: Research agenda for frailty in older adults: toward a better understanding of physiology and etiology: summary from the American Geriatrics Society/National Institute on Aging Research Conference on Frailty in Older Adults. *J Am Geriatr Soc.* 2006;54(6):991–1001. 10.1111/j.1532-5415.2006.00745.x 16776798

[ref-50] Warner HR Sierra F: The longevity dividend: why invest in basic aging research? *Can J Aging.* 2009;28(4):391–4; French 395–8. 10.1017/S0714980809990286 20166274

[ref-51] Westbrook R Bonkowski MS Strader AD: Alterations in oxygen consumption, respiratory quotient, and heat production in long-lived GHRKO and Ames dwarf mice, and short-lived bGH transgenic mice. *J Gerontol A Biol Sci Med Sci.* 2009;64(4):443–51. 10.1093/gerona/gln075 19286975 PMC2657169

[ref-52] Wilkinson JE Burmeister L Brooks SV: Rapamycin slows aging in mice. *Aging Cell.* 2012;11(4):675–82. 10.1111/j.1474-9726.2012.00832.x 22587563 PMC3434687

[ref-53] Zhang Y Bokov A Gelfond J: Rapamycin Extends Life and Health in C57BL/6 Mice. *J Gerontol A Biol Sci Med Sci.* 2014;69(2):119–30. 10.1093/gerona/glt056 23682161 PMC4038246

[ref-54] Zhou Y Xu BC Maheshwari HG: A mammalian model for Laron syndrome produced by targeted disruption of the mouse growth hormone receptor/binding protein gene (the Laron mouse). *Proc Natl Acad Sci U S A.* 1997;94(24):13215–20. 9371826 10.1073/pnas.94.24.13215 PMC24289

