# Supplementary material for: Interaction of growth hormone receptor/binding protein gene disruption and caloric restriction for insulin sensitivity and attenuated aging
Source: F1000Res. 2014 Oct 28;3:256. [Version 1] doi: 10.12688/f1000research.5378.1 (PMC4358413; doi:10.12688/f1000research.5378.1)
Supplement: Peer review history

# Peer review: Referee response for version 1

**DOI:** 10.5256/f1000research.5744.r7783

**Published:** 2015-03-12

**Authors:** Florian Muller

**Affiliations:** 1 Department of Genomic Medicine, University of Texas MD Anderson Cancer Center, Houston, TX, USA

The study addresses a long running question in aging research, which concerns the interaction of the two regimes known to consistently extend mammalian (mouse) lifespan: caloric restriction and manipulation of the GH/IGF1 axis.

The study provides a wealth of very useful endocrinological and metabolic data and the authors must be commended their extensive work. The interpretation with regards to the effects of insulin sensitivity and caloric restriction are plausible, though ultimately, whether this is truly the mechanism by which the lifespan-extending effects are mediated (and account for the differential response to CR in GHRko mice), will require direct experimental demonstration.

I would like the authors to discuss the metabolic results a little more. For example, it is indicated that the total caloric output of GHrKO mice is higher, considerably so, than WT (regardless of CR). However, the heat production is more or less the same. At the same time, CR has opposite effects on hear production in GHRko versus WT mice. It would seem to me overall, that as a whole the metabolic effects of CR in table 3, are much attenuated in the GHRko mice, i.e. WT mice have much more profound alterations in metabolic parameters in response to CR than do GHRko mice to CR.

These are just suggestions. Overall, the data and paper are very valuable as is.

I have read this submission. I believe that I have an appropriate level of expertise to confirm that it is of an acceptable scientific standard.

# Peer review: Referee response for version 1

**DOI:** 10.5256/f1000research.5744.r6555

**Published:** 2014-11-18

**Authors:** Subburaman Mohan

**Affiliations:** 1 Department of Physiology and Pharmacology, Loma Linda University, Loma Linda, CA, 92357, USA

Caloric restriction prolongs longevity in experimental animals via altering insulin sensitivity. The growth hormone receptor knockout (GHR KO) mice live longer and have increased insulin sensitivity. In this study, the authors tested the hypothesis that the modest effect of caloric restriction on longevity of GHR KO mouse is because of the failure of caloric restriction to further increase the already maximally elevated insulin sensitivity in this mouse model.  Surprisingly, the authors found that caloric restriction increased blood insulin concentration and reduced insulin sensitivity in GHR KO mice. These findings refute the established assertion between insulin sensitivity and and slowed senescence in GHR KO mice  The paper is well written and the data analyses are appropriate. The conclusions are supported by experimental data. The number of animals per group are considerably large except for GHR-N on caloric restricted group. I recommend indexing the paper as submitted.

I have read this submission. I believe that I have an appropriate level of expertise to confirm that it is of an acceptable scientific standard.
